# Estrogen receptor-α is localized to neurofibrillary tangles in Alzheimer’s disease

**DOI:** 10.1038/srep20352

**Published:** 2016-02-03

**Authors:** Chunyu Wang, Fan Zhang, Sirui Jiang, Sandra L. Siedlak, Lu Shen, George Perry, Xinglong Wang, Beisha Tang, Xiongwei Zhu

**Affiliations:** 1 Department of Neurology, the second Xiangya Hospital, Central South University, Changsha, Hunan, People’s Republic of China; 2 Department of Pathology, Case Western Reserve University, Cleveland, Ohio, USA; 3 Department of Neurosurgery, Chengdu first people’s Hospital, Chengdu, The People’s Republic of China; 4 Department of Neurology, Xiangya Hospital, Central South University, Changsha, Hunan, People’s Republic of China; 5 Department of Biology, The University of Texas at San Antonio, San Antonio, Texas, USA

## Abstract

The female predominance for developing Alzheimer disease (AD) suggests the involvement of gender specific factor(s) such as a reduced estrogen-estrogen receptor signaling in the pathogenesis of AD. The potential role of ERα in AD pathogenesis has been explored by several groups with mixed results. We revisited this issue of expression and distribution of ERα in AD brain using a specific ERα antibody. Interestingly, we found that ERα co-localized with neurofibrillary pathology in AD brain and further demonstrated that ERα interacts with tau protein *in vivo*. Immunoprecipitaion experiments found increased ERα-tau interaction in the AD cases, which may account for ERα being sequestered in neuronal tau pathology. Indeed, tau overexpression in M17 cells leads to interruption of estrogen signaling. Our data support the idea that sequestration of ERα by tau pathology underlies the loss of estrogen neuroprotection during the course of AD.

Alzheimer’s disease (AD) is a progressive neurodegenerative disorder characterized by selective neuronal loss in the hippocampus and temporal cortex along with distinctive hallmark pathologies such as neurofibrillary tangles (NFTs) and senile plaques^1^,^2^.

Multiple studies demonstrated that the incidence and prevalence of AD is higher in women than in men^3^,^4^. The female predominance for developing AD suggests the involvement of gender specific factor(s) in the pathogenesis of AD. Estrogen, which belongs to the category of neurosteroids, appears to be involved in cognition and memory as well as sexual behavior^5^,^6^. There is a loss of cognitive enhancing effect of estrogen with advanced age likely linked to age-related changes in the expression and signaling of the estrogen receptors (ERs)^7^. Not surprisingly, estrogen loss in menopausal women has been suspected to be closely related with the occurrence of AD^8^. Along this line, some clinical and experimental studies supported a preventive role of estrogen replacement therapy in cognitive deterioration-related disease, such as AD^9^, although it should be noted that several other studies failed to replicate such beneficial effects^10^. Estrogen acts as a neuroprotectant by reducing toxicities of glutamate and amyloid beta (Aβ)^11^, by enhancing synaptic plasticity, regulating neurotrophic factors, facilitating transcription factor activation, reducing inflammation in the brain^12^,^13^ , and by decreasing the hyperphosphorylation of tau^14^,^15^,^16^,^17^.

Estrogen response is mainly mediated by the nuclear receptor family of transcription factors, estrogen receptor α (ERα) and estrogen receptor β (ERβ)^18^,^19^ and the newly described G protein-coupled estrogen receptor 1^20^. Estrogen-bound ERs translocate to the nucleus, where they increase transcription of target genes that likely underlies most of the neuroprotective effects^21^. ERα and ERβ are both widely distributed in the brain^22^, and ERα mediates a stimulatory, while ERβ mediates an inhibitory, effect of estrogens^23^. Indeed, ERα appears more effective than ERβ in inducing transcription linked to the estrogen response element (ERE)^24^,^25^,^26^,^27^,^28^. Long-term ovariectomy led to a significant decrease of hippocampal ERα, but not ERβ, in rats^29^,^30^,^31^. Treatment with 4,4’,4″-(4-propyl-[1H]-pyrazole-1,3,5-triyl) trisphenol (PPT), an ERα -specific agonist, improved memory impairment in ovariectomized rats^32^. Human studies involving polymorphisms in the ERα gene reveal the importance of ERα receptors in maintaining memory function^33^,^34^,^35^,^36^,^37^. The potential roles of ERα in AD pathogenesis have been explored by several groups with mixed results: More nuclear ERα expression was found in the nucleus basalis of Meynert^38^, diagonal band of Broca^39^, medial mammillary nucleus^40^, infundibular nucleus of hypothalamus^41^ in AD patients than in sex- and age-matched controls and it was suggested that such increased nuclear ERα likely predispose to reduced activity and increases the risk of these neurons to develop AD neuropathology; In marked contrast, decreased total, and more importantly, nuclear ERα, was found in pyramidal neurons of hippocampus in AD patients^42^, suggesting an important neuroprotection role of nuclear ERα in AD. Similarly controversial, while estrogen decreases the hyperphosphorylation of tau in a ER-dependent manner^43^ supporting the observation that females have more affected brain regions with NFTs than males^44^ and show more severe early tau alterations in the nucleus basalis of Meynert^45^, a recent study demonstrated that overexpression of ERα caused tau hyperphosphorylation and aggregation^46^.

In this study, we revisit the issue of expression and distribution of ERα in specific AD brain regions (i.e., hippocampus and cortex) with a focus on its relationship with tau alterations. While previous reports used a C-terminal antibody, we used a specific antibody recognizing an N-terminal region of ERα, and found that ERα co-localized with NFT in AD hippocampus and cortex. Further, by immunodot and immunoprecipitation assays we found an increased ERα-tau interaction in the AD brain which likely resulted in large amount of ERα being sequestered in the paired helical filaments (PHF) and neuritic tau pathology in the AD brain. Further, with a cell based luciferase assay, we show that indeed tau overexpression leads to interruption of estrogen signaling. Our data appear to suggest that the sequestration of ERα by PHFs underlies the loss of estrogen neuroprotection in those areas vulnerable to neurodegeneration such as the hippocampus and cortex during the course of AD.

## Results

### IHC analysis of ERα in AD hippocampus and cortex

**Figure 1:**
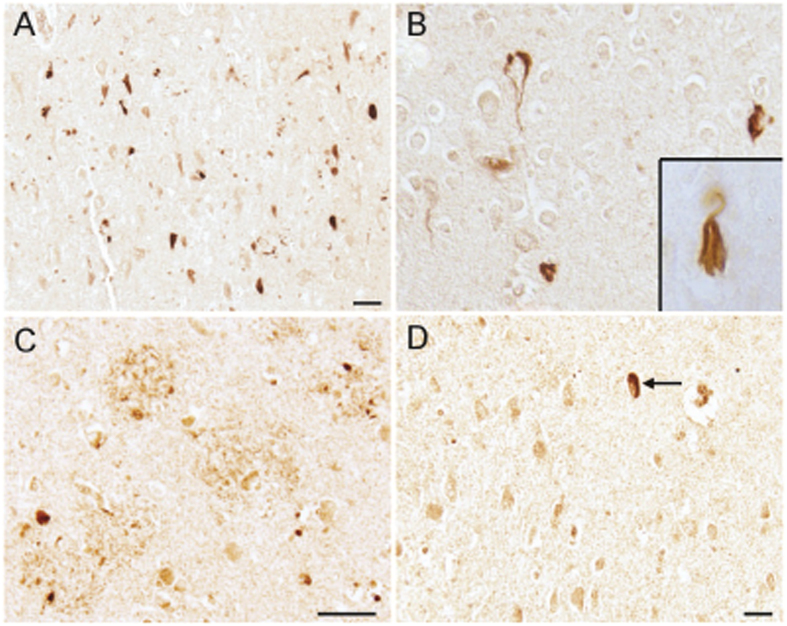
In the hippocampus, ERα is localized to neurons with neurofibrillary tangle-like morphology in cases of AD (A). At higher magnifications neurons with the appearance of both intracellular NFT (**B**) and extracellular NFT (B, inset) contain ERα. Other neurons have a weak cytoplasmic stain. Dystrophic neurites surrounding amyloid plaques are also stained (**C**). In age- and gender-matched controls (**D**), weak neuronal cytoplasmic staining and occasional strongly stained aging-associated NFTs (arrow) are present. Scale bars =  50 μm.

In the hippocampal and cortical tissues of all AD cases examined, neuronal staining for ERα was a prominent finding, both with a weak cytoplasmic stain, but often specifically with a fibrillar appearance suggesting ERα was in neurofibrillary tangles (Fig. 1A), also shown at a higher magnification (Fig. 1B). Inset of Fig. 1B shows a representative neuron with fibrillar extracellular NFT-like morphology stained for ERα. In addition, dystrophic neurites surrounding senile plaques were also labeled (Fig. 1C). In age-matched control individuals, weak neuronal cytoplasmic staining was seen and the rare age-associated NFTs (arrow) were also well stained (Fig. 1D).

**Figure 2:**
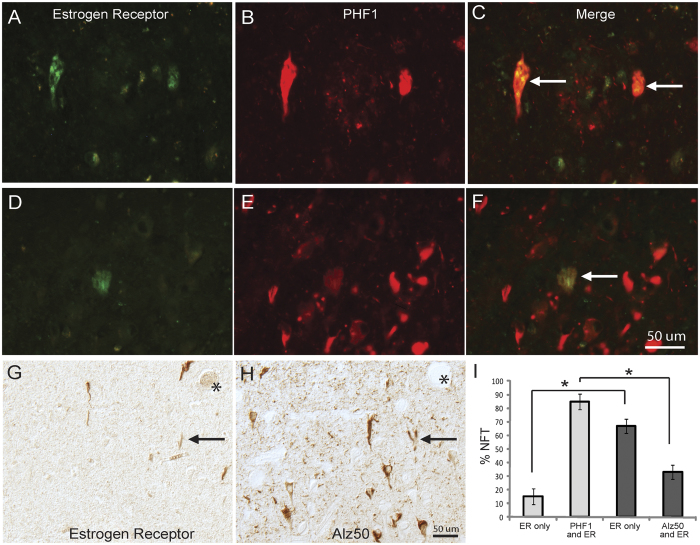
ERα co-localizes with NFT in the hippocampus. (**A–F**) Double fluorescence immunocytochemistry shows that NFTs stained for ERα (green) (**A,D**) most often also contain tau protein phosphorylated at Ser396/404 identified by PHF-1 antibody (red) (**B,E**). (**C,F**) showed the merged images. Two separate fields show how only a subset of PHF-1-positve NFT contain ERα (arrow in C and F). Using light level DAB staining of serial adjacent sections, ERα-NFT were directly compared with Alz-50, which detects a conformational epitope of tau (G,H, *denotes landmark vessels and arrow show the same neurons in each section). Quantification of 3 AD cases using this method found that PHF-1 colocalizes with more ER-positive NFTs than Alz-50 (I, *p < 0.001). One way ANOVA with Newman-Keuls test.

To confirm the localization of ERα in NFTs, double fluorescence staining with antibodies to ERα and hyperphosphorylated tau (PHF-1) revealed that ERα-positive NFTs were also positive for PHF-1 (Fig. 2A–F). To quantify the population of NFT containing ERα, adjacent serial sections were stained using Alz-50 as a relative early marker for NFT^47^,^48^, PHF-1 as a relative late marker for NFT^49^, and ERα, and the numbers of NFT stained for one or both antibodies were counted.

**Figure 3:**
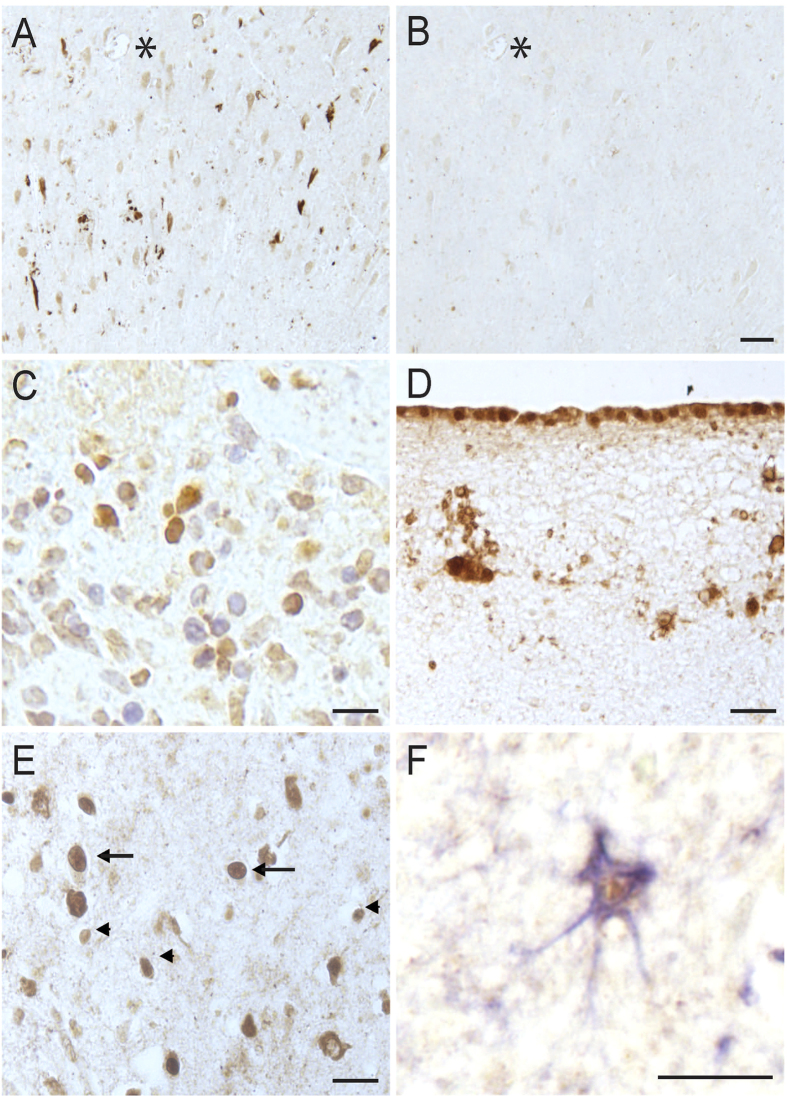
Specificity of the ERα antibody. Adsorption of the ERα antibody with its peptide antigen abolished the neuronal and NFTs immunostaining in the hippocampus (**A,B**, *denotes landmark vessel on adjacent sections, Scale bar = 50 μm). A sample of breast cancer tissue revealed positive nuclear signal for the antibody as expected (**C**). The specificity of the antibody was further confirmed as the pia mater and glial cells near the outermost edge of each hippocampal tissue section in all cases was stained as has been previously shown (Scale bar C–F = 20 μm). In addition to glial nuclei staining found in the hippocampus of every case examined (arrowheads, **E**), occasional neuronal nuclei were found to contain ERα (**E**, arrows). Many GFAP-lableled astrocytes had ERα nuclear staining (**F**, GFAP-blue, ERα- brown).

Comparing to either Alz-50 or PHF-1 staining, only a small portion (25-35%) of NFTs were positive for ERα imunoreactivity since many more NFTs were only positively stained by Alz-50- or PHF-1, respectively. It was interesting to note that the majority (≈85%) of ERα-positive NFTs were also PHF-1-postive with only very few NFTs only positive for ERα (≈15%) (Fig. 2I). In contrast, there was significantly fewer ERα-positive NFTs co-stained with Alz-50 (33%, *p* < 0.001) while the majority of ERα-positive NFTs contained no Alz-50 immunoreactivity (Fig. 2G–I). In the hippocampus and cortex, only some cases revealed neurons with clear nuclear ERα staining that is much darker than the cytoplasmic staining (4 of 12 AD cases and 2 of 6 controls) (Fig. 3E arrows) and no apparent difference in the density of neurons with nuclear ERα staining between AD and control was noted. These neurons are almost always devoid of NFTs in AD. No correlation of either nuclear staining patterns or intensity was found with age, gender, or postmortem interval.

To confirm the specificity of the antibody, adsorption of the rabbit polyclonal antibody revealed nearly complete reduction of the neuronal immunoreactivity as seen in adjacent serial sections (Fig. 3A,B, *denotes landmark vessel). Also, in breast cancer tissue sections used as a positive control, positive nuclear staining was found as expected (Fig. 3C). In all cases examined for this study, both AD and control, portions of the outermost layer of the tissue section, likely the pia mater, was prominently stained, with nearby glial cells also strongly labeled (Fig. 3D). This staining pattern has been demonstrated in rat brain^50^,^51^ and thus the similar finding here in human brain further confirms the specificity of the antibody. It has been reported that ERα is present in glial cells^52^, and indeed throughout the hippocampus in all cases of both AD and control groups used in this study, small nuclei were readily stained for ERα, representing a glial cell population (arrowheads, Fig. 3E). Double staining found many astrocytes with ERα in the nuclei (Fig. 3F, GFAP-blue, ERα-brown). Yet, no difference in the number of ERα-positive astrocytes was noted between AD and control.

### Western blot analysis of ERα in AD brain

**Figure 4:**
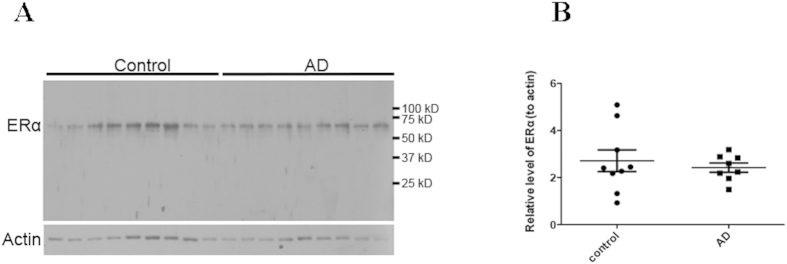
Western blot analysis of ERα in AD and control. (**A**) Representative immunoblot of cortical gray matter, homogenized in lysis buffer and probed with antibody against ERα, show a strong band at the expected molecular weight of 66 kD in AD and control samples (n = 9/group). β-actin was used as an internal loading control. (**B**) Quantification of ERα immunoblots, which is normalized to actin levels, shows no significant difference of ERα levels between AD and control. Gels were prepared and run in triplicate and data are means ± s.e.m of these three independent experiments. Student t test.

Western blot analysis of the soluble fractions from cortical homogenates from brains of nine AD patients and nine age-matched controls revealed a single specific band at the expected molecular weight of 66 kD for ERα (Fig. 4A), which further confirmed the specificity of the ERα antibody used in this study. Though the control cases showed greater variability of ERα, and the AD had more consistent levels, quantification found there was no overall decrease of ERα in AD (*p* > 0.05; Fig. 4B).

### ERα is closely associated with tau

**Figure 5:**
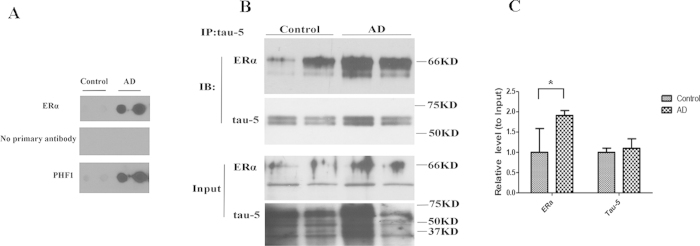
Interaction of ERα with tau. Dot blot assay of paired helical filaments (PHFs), purified from AD and controls cases and probed with ERα antibody show strong immunoreactivity for PHFs (A). PHF-1 (positive control) and omission of primary antibody (negative control) are also shown (**A**). (**B**) Cortical tissues from AD and control were lysed by RIPA buffer and immunoprecipitated (IP) with tau-5 antibody. Tau-5 immunoprecipitates (upper panel) and input (lower panel) were analyzed by immunoblot (IB) with antibodies against ERα and tau-5. (**C**) Quantification of (**B**) revealed that significantly more ERα was co-immunoprecipitated with tau-5 antibody in AD brain lysates (n = 2) than in control brain lysates (n = 2). Data are means ± s.e.m of three independent experiments. *p < 0.01, with representative blots from one experiment shown. Student t test.

The nearly complete overlap of ERα and PHF-1 in NFTs suggests that ERα may be a component of PHF of NFTs. Since the PHF component of NFTs is very insoluble, we next sought to determine whether ERα is present in the insoluble PHF fractions prepared from human brain by using a dot-blot assay.10 ug samples of enriched PHF fractions purified from cortical tissue of AD cases and controls were stained for PHF-1 and ERα. Most PHF fractions prepared from control patient brain tissue showed little to no PHF-1 immunoreactivity, however, in those control cases with hyperphosphorylated tau, some ERα was also present. PHF prepared from AD brain tissues demonstrated strong immunoreactivity for both PHF-1 and ERα immunoreactivity (Fig. 5A), which not only confirms the presence of ERα in the PHFs, but also demonstrates more ERα is likely sequestered in AD brain.

The co-purification of PHF and ERα prompts us to explore whether there is direct interaction between ERα and tau protein, the major protein in PHF, by co-immunoprecipitation experiment. An antibody against total tau (i.e., tau-5) was used to immunoprecipitate tau protein from cortical homogenates from cases of AD and age-matched normal controls and the tau-5 immunoprecipitates were probed by ERα antibody. Indeed, significant amounts of ERα were pulled down by tau-5 antibody in the brain homogenates which confirmed a physical interaction *in vivo* under physiological condition (Fig. 5B). Moreover, more ERα was found in the tau-5 immunoprecipitates from AD brain than control brain (*p* < 0.05) (Fig. 5B,C), demonstrating increased interaction between tau and ERα in the AD brain which is likely the mechanism that underlies increased sequestration of ERα by the PHFs in NFTs.

### Tau overexpression inhibits transcriptional activity of ERα

**Figure 6:**
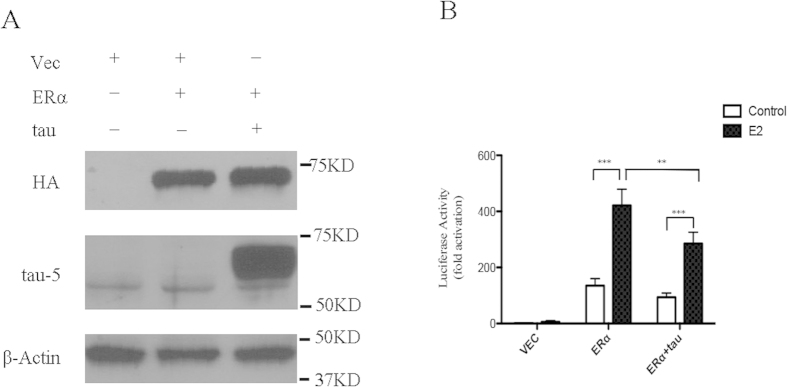
Luciferase assay demonstrated that tau overexpression inhibited E2-stimulated transcriptional activity of ERα in M17 cells. (**A**) Immunoblot analysis of ERα (HA-tagged) and tau in M17 cells transiently transfected with indicated vectors. (**B**) Tau overexpression reduces E2-stimulated transcriptional activity of ERα by 30%. The ERE reporter luciferase activity was normalized against the control renilla luciferase activity to correct for transfection efficiency. All values were presented as the fold induction over the control luciferase activity in the untreated vector cells, which was taken as 1. Data are means ± s.e.m of three independent experiments. **p < 0.01, ***p < 0.001. One way ANOVA with Newman-Keuls test.

The potential sequestration of ERα by tau protein through direct interaction prompted us to explore whether tau expression has any effects on the transcriptional activity of ERα in neurons using a dual luciferase assay. M17 human neuroblastoma cells were transiently transfected with vector, ERα, or both ERα and human tau htau40 together with the 3xERE-TATA-luc and a renilla luciferase reporter, pRL-SV40-Luc, as an internal control for transfection efficiency. Western blot analysis confirms the overexpression of ERα and tau (Fig. 6A). Without ERα overexpression (i.e., vector only), there was minimal estrogen-responsive reporter 3xERE-TATA-luc activity in the absence or presence of E2. Treatment of ERα or ERα + tau transfected M17 cells with E2 resulted in a significantly differential increase in the 3xERE-TATA-luc activity compared with the basal level in non-E2 treated cells (*p* < 0.001) (Fig. 6B). Overexpression of the tau protein in M17 cells (i.e., ERα+tau) significantly decreased E2-stimulated reporter activity by 30% compared with the ERα only cells (*p* < 0.01) (Fig. 6B).

## Discussion

In this study, we focused on the distribution of ERα as it relates to tau alterations in the hippocampus and cortex of AD patient brains and reported several novel findings: 1) for the first time, we found that ERα is localized to neurofibrillary tangles in AD brain with nearly complete overlap with hyperphosphorylated tau as marked by PHF-1 antibody; 2) there is no significant difference in the expression of ERα between AD and control as shown by western blot analysis; 3) ERα co-purifies with paired helical filaments (PHFs) prepared from AD brain homogenates; 4) ERα interacts with tau proteins *in vivo* and there is increased ERα-tau interaction in AD brain. To pursue the functional consequence of increased ERα and tau interaction in AD, we further explored the effects of tau expression on ERα signaling in M17 cells and found that tau overexpression inhibits the transcriptional activity of ERα.

The cellular location of ERα is very important for its role as a transcription factor: Under unliganded condition, ERα exists in a monomeric form complexed with heat shock proteins and is distributed between the nucleus and cytoplasm. Upon binding to its ligand, ERα dissociates from HSPs, dimerizes, and translocates to the nucleus, where it interacts with co-activator complexes and regulates the expression of target genes. It was previously demonstrated by immunocytochemistry that hippocampal ERα expression is decreased in AD^42^,^53^, but the expression of the wild type ERα mRNA is not changed in the temporal cortex of AD patients^54^. Paradoxically, nuclear ERα was increased in the nucleus basalis of Meynert, diagonal band of Broca, medial mammillary nucleus, infundibular nucleus of hypothalamus^38^,^39^,^40^ but reduced in pyramidal neurons in the hippocampus of AD patients^41^,^42^. These studies appear to suggest a brain region-specific regulation of the expression and distribution of ERα and it was believed that ER splice variants may be involved^53^,^54^. More detailed studies to confirm these observations and explore the underlying mechanisms are obviously needed.

The current study focuses on the expression and distribution of ERα in the hippocampal and cortical areas in AD. Our results are very similar to previous findings such that only a small number of pyramidal neurons display nuclear ERα immunoreactivity^42^, but no apparent difference in the nuclear staining between AD and control was noted in our study. We were able to perform western blot with this antibody which revealed no significant difference in the levels of soluble ERα between AD and control cortical samples. Of course, this does not preclude the possibility that ERα levels may vary in different cell types or in other brain regions. The most significant and novel finding of our study is that ERα co-localized with NFTs in AD brains as demonstrated by both immunocytochemistry and dot blot. Although it was previously reported that some pyramidal neurons contain both ERα and Alz-50 immunoreactivity in the hippocampus from AD patients^42^, this is the first report to provide evidence that ERα actually co-localizes with NFTs. Such an observation is unlikely an artifact since we demonstrated that the antibody works as expected in the control experiments and most importantly, it specifically recognize one single band without any cross-reactivity with known tau species in AD brain tissues. It must be noted that the antibody used in the present work is against an N-terminal epitope of ERα, while the previous studies used a C-terminal specific antibody^42^,^53^. Major hippocampal splice isoforms of ERα include MB1^53^ which lacks 168nt in the exon 1 encoding the ligand-independent transactivation function (AF)-1 within the N-terminal and the antibody used in this study may not recognize the resulting MB1 protein. It is not unlikely, therefore, that the differences noted between our study and the prior study on hippocampus may in fact reflect splice variants of ERα in the brain. Indeed, besides finding that some splice variants are lower in AD, and thus may not be able to reduce estrogen signaling, the regional specific changes in splice variants found in AD^53^,^54^ may be one contributing factor to the systematic progression of the disease through the different brain regions^54^. It is interesting that gender differences are seen in ERα localization in some brain regions including the hypothalamus, yet in the hippocampus and cortex, both males and females show similar levels of NFT immunolocalization.

Interestingly, our detailed co-staining analysis of the population of NFTs containing ERα revealed that ERα co-localized with PHF-1-positive NFT more often than with the Alz-50-positive NFT. PHF-1 detects hyperphosphorylated tau and Alz-50 labels misfolded tau, often considered an early tau change in AD^47^,^48^. Our results demonstrated that ERα is likely more tightly associated with (hyper)phosphorylated tau protein rather than conformational-abnormal tau. The interaction between tau and ERα as demonstrated by the co-immunoprecipitation experiments provides the biochemical basis for the localization and sequestration of ERα by PHFs in the AD neurons. Along with increased phosphorylation and deposition of tau in AD brain, there is also increased ERα being co-purified with PHFs or co-immunoprecipitated by tau-5 antibody in AD brain, demonstrating increased sequestration of ERα by NFTs in the pyramidal neurons in AD brain which likely limits the bioavailability of functional ERα and results in functional consequences. In this regard, it was noticed in our study that no neurons containing ERα-positive NFTs demonstrated any nuclear ERα immunoreactivity. Consistently, a similar observation was reported previously demonstrating that ER-α was only located in the cytoplasm in those neurons with both ERα and Alz-50 immunoreactivity in AD brain^42^. Along this line, we further demonstrated that tau overexpression leads to 30% inhibition of E2-stimulated transcriptional activity of ERα in M17 cells, thus confirming the negative effects of increased tau-ERα interaction on ERα signaling and neuroprotection in the pyramidal neurons in AD brain. Our results implicate that the great variation in tau pathology among AD patients could underlie the inconsistent results of the neuroprotection study of estrogen. Given that the current study focused on hippocampal and cortical regions, it must be emphasized whether our observation and conclusion can be generalized needs further study in other brain regions. Nevertheless, it was of interest to note that tau expression was proposed as an effective predictor for breast cancer sensitivity to taxanes-based neoadjuvant chemotherapy, especially in the ER+ subgroups^55^,^56^,^57^,^58^, our study could shed new light on the molecular mechanism underlying the utility of tau for individualizing adjuvant chemotherapy in breast cancer patients.

## Methods and Materials

### Immunohistochemistry

Samples of hippocampus, obtained at autopsy and following approved IRB protocols, were received from the Brain Bank at Case Western Reserve University in accordance with the institutional bioethics guidelines. The diagnosis of Alzheimer’s disease was obtained according the NINCDS-ADRDA group criteria^59^. Samples were fixed in buffered formalin or methacarn (methanol: chloroform: acetic acid; 6:3:1), embedded in paraffin and serial adjacent sections were cut. Histopathologically diagnosed cases of AD (n = 12, half male and half female, age 78.1 ± 1.5, postmortem interval of 13.3 ± 3.7 h) and similarly aged control cases (n = 6, half male and half female, age 72.5 ± 3.9, postmortem interval of 14.2 ± 3.3 h) were used. Equal numbers of males and females were examined. Paraffin embedded tissue sections were deparaffinized and rehydrated by 10 min each treatment in 2 changes of xylene followed by 100%, 95%, 70%, 50% ethanol, and finally to Tris buffered saline (50 mM Tris, 150 mM NaCl, pH = 7.6). Antigen retrieval by pressure cooking in acetate buffer decloaker (Biocare Medical) was used for all staining experiments. After blocking in 10% normal goat serum (NGS) in TBS for 30 min, primary antibodies were applied and incubated for 16 hr at 4 °C. Antibodies used included rabbit anti-alpha estrogen receptor (Abcam, ab131437), rabbit or mouse anti-GFAP (Invitrogen and clone GA5) and mouse anti-tau antibodies Alz-50^60^ (gift of Dr. Peter Davies, Albert Einstein College of Medicine, Bronx NY, USA) and PHF-1^61^ (gift of Dr. Sharon Greenberg and Dr. Peter Davies). The peroxidase-anti-peroxidase method was used and developed using DAB (Dako). To compare the immunostaining pattern of ERα with the glial marker GFAP, or with markers of neurofibrillary tangles Alz-50 recognizing a conformational epitope of tau requiring both N-terminal amino acid sequence 7–9 amd C-terminal region 312–342 (considered an early tau marker) and PHF-1 recognizing ta phosphorylated at Ser396/404 (considered a later tau marker) either double stain light immunocytochemistry using Fast Blue as chromagen or double label fluorescence using Alexafluor 488 or 568 labeled secondary antibodies and imaged with a Zeiss Axiocam was performed. Using the light level method any autofluorescence present in autopsy brain tissue is corrected for, yet both methods confirmed the staining patterns and gave very similar results.

To confirm the specificity of the rabbit ERα antibody, diluted antibody was incubated with 5 micrograms of peptide (Abcam) for 16 hours and then applied to tissue section. On the adjacent section, unadsorbed antibody was applied.

To quantify the number of NFTs stained by ERα in the hippocampus, 3 adjacent fields of the CA1 region stained for ERα, and then by PHF-1 or Alz-50 were imaged. Following tissue landmarks, individual NFTs present in each field were counted as either stained by one antibody or by both antibodies. Quantification was performed on 3 cases of Alzheimer disease and one way ANOVA with Newman-Keuls test was used to compare the % of ERα-positive NFT colocalized with either Alz-50 or PHF-1. Further, double stained sections were also examined to further compare the staining patterns qualitatively.

### Western blotting

Samples of frozen cortical gray matter of AD and age- and gender-matched control cases (n  =  9/group) were homogenized and lysed with RIPA Buffer (Cell Signaling) plus 1 mM phenylmethylsulfonyl fluoride (Sigma) and Protease Inhibitor Cocktail (Sigma) and centrifuged for 10 min at 16,000 × g at 4 °C Protein concentrations of the lysates from total cortical gray matter homogenates were determined by the bicinchoninic acid assay method (Pierce, Rockford, IL, USA) Equal amounts of proteins (10 μg) were separated by sodium dodecyl sulfate polyacrylamide gel electrophoresis (SDS-PAGE) and transferred to immobilon membranes. After blocking with 10% non-fat dry milk, primary and secondary antibodies were applied and the blots developed with enhanced chemiluminescence (ECL) (Millipore). β-actin was used as a gel loading control for cortical gray matter homogenates. Antibodies used included ERα and anti-β-actin (Millipore). The gels were run and stained in triplicate. All blots and dot blots were scanned at high resolution and the immunoreactive bands and dots were quantitated with Image J software.

### Dot blot

Dot-blot assay was applied to determine the levels of ERα present in enriched insoluble PHF fractions. The PHFs were purified from the hippocampus and temporal cortex of AD and age- and gender-matched control cases (n = 7/group) as previously published^62^. For each case, 10 ug samples of PHFs suspended in 50 ul of PBS were applied to immobilon membrane in duplicate using a Bio-Dot Apparatus (Bio-Rad, USA). The samples were allowed to bind to the membrane overnight at 37 °C and then vacuum dried for 10 min. At this point the membrane was removed from the dot-blot apparatus and allowed to airdry completely. The dot blot was then wetted with methanol, rinsed in PBS and blocked with 10% non-fat dry milk in PBS-T. After incubation with anti-PHF-1 or anti-ERα overnight at 4 °C, the dot blot was washed three times, incubated with the corresponding secondary antibody for 1hr at room temperature, washed again three times, and exposed using ECL. The primary antibody was omitted as a negative control.

### Co-immunoprecipitation (Co-IP)

To determine whether tau interacts with ERα, Co-IP analysis was conducted, using cortical protein lysates from postmortem brain tissues of AD patients and matched control subjects. Samples of frozen cortical gray matter of AD and control were homogenized in RIPA buffer containing protease inhibitors (Cell Signaling) on ice and centrifuged for 10 min at 16,000 × g at 4 °C. 500 ug of lysate was incubated with 20 ul of tau-5 antibody (Abcam) using Dynabeads® Protein G—CoIP Kit (Invitrogen) following the manufacturer’s protocol. Negative controls included IP with normal mouse IgG (Cell Signal). Input and IP samples were run on SDS-PAGE and western blot was performed using the ERα antibody and tau-5.

### Cell Culture, Transfection, Treatment and Luciferase activity assay

M17, a widely used human neuroblastoma cell line, was maintained in Opti-MEM medium (Invitrogen, Eugene, OR), supplemented with 10% fetal bovine serum and 1% penicillin-streptomycin, in a humid incubator with 5% carbon dioxide at 37 °C. Transfection was performed using lipofectamine 2000 following the instructions (Invitrogen). For the co-transfection experiments, the plasmid DNA ratio of vitellogenin Estrogen Response Element (3X ERE TATA luc) (Addgene) to expression vectors of ER-α or/and tau was 1:2. A Renilla luciferase reporter, pRL-SV40-Luc (Promega), was used as an internal control for transfection efficiency. After transiently co-transfecting ERE and Renilla (VEC), or ERE, Renilla and pcDNA-HA-ERα (Addgene) (ERα) or ERE, Renilla, pcDNA-HA-ER and pCI-neuro-tau40 (gift of Chengxin Gong) (ERα + tau), cells were treated and incubated for 24 h with 10 nM 17β-Estradiol (Sigma) in Opti-MEM medium with 2% fetal bovine serum and 1% penicillin-streptomycin. 17β-Estradiol was dissolved in 100% DMSO in appropriate concentrations; stock solutions were a 1000-fold of the desired concentration in the medium. DMSO was used as vehicle control for all experiments. Luciferase activities in total cell lysate were measured using the Dual Luciferase Assay System (Promega) according to the manufacturer’s instructions in a FluorStar OPTIMA (BMG). Absolute ERE promoter firefly luciferase activity was normalized against Renilla luciferase activity to correct for transfection efficiency and expressed as relative fold change in luciferase activity relative to the empty vector vehicle-treated control condition. Triplicate wells were assayed for each transfection condition, and at least three independent transfection assays were performed.

### Statistical Analysis

Statistical analysis was performed using the SPSS 17.0 software. Data with two groups were analyzed by Student’s t-test. Data with more than two groups were processed with one way analysis of variance (ANOVA) with Newman-Keuls test. All the data were expressed as means ± SEM. p < 0.05 was considered as statistically significant.

## Additional Information

**How to cite this article**: Wang, C. *et al*. Estrogen receptor-α is localized to neurofibrillary tangles in Alzheimer’s disease. *Sci. Rep*. **6**, 20352; doi: 10.1038/srep20352 (2016).
